# Comparative Evaluation of the Antibiotic Resistance Profile of *Staphylococcus aureus* Isolated From Breeders and Livestock

**DOI:** 10.3389/ijph.2024.1607603

**Published:** 2024-09-18

**Authors:** A. C. Ifediora, E. Enya, C. S. Mbajiuka

**Affiliations:** Department of Microbiology, College of Natural Sciences, Michael Okpara University of Agriculture Umudike, Umudike, Abia State, Nigeria

**Keywords:** poultry, *Staphylococcus aureus*, livestock, resistance, prevalence

## Abstract

**Objectives:**

Animals are a potential source of Methicillin Resistant *Staphylococcus aureus*. This study evaluated the antibiotics susceptibility pattern of *S. aureus* isolates from breeders and livestock.

**Methods:**

*S. aureus* strains were isolated from 180 livestock and 48 livestock farmers and identified using standard methods. Antibiotic susceptibility profiles and MRSA status were determined via disk diffusion susceptibility method.

**Results:**

Among farm workers, 37.5% were colonized by *S. aureus*, with pig farm workers exhibiting the highest prevalence (56.2%), cattle herders (37.5%), and goat farm workers (18.7%). MRSA carriage among livestock isolates was 41.3%, while, six isolates from the poultry farm worker were MRSA, representing a carriage of 33.3%. Drug susceptibility profiles revealed differential patterns between isolates from breeders and animals. Gentamicin and levofloxacin demonstrated higher efficacy against farm worker isolates compared to animal isolates. Resistance to cefuroxime was higher among animal isolates (84.1%) as against the 66.7% for the breeders.

**Conclusion:**

The identification of multidrug-resistant *S. aureus* strains underscores the risk posed to humans in contact with animals. These findings stress the importance of monitoring and managing MRSA transmission between animals and humans.

## Introduction

*Staphylococcus aureus* is a distinct microorganism that is well-known worldwide for its clinical significance in causing mastitis in livestock [1] as well as a number of infection in humans, such as bacteremia, endocarditis, and infections of the skin and soft tissues. There have been reports of this organism’s spread across other animal species, particularly in areas with a high concentration of livestock farming, as well as that of its variation, methicillin-resistant *S. aureus* (MRSA).

Nigeria is an agrarian country where raising livestock is a significant source of income. In addition to commercial farms, subsistence poultry farming is also carried out. Therefore, it may not be possible to completely eradicate MRSA from the animal population. Instead, it is strongly recommended to investigate ways to stop the spread of animal Associated MRSA (LA-MRSA), particularly among livestock workers. According to Khairullah et al. [2], animals have emerged as a significant secondary source of MRSA in the environment, and human-animal contact frequently serves as a major conduit for the spread of the bacteria.

Controlling the worldwide silent antimicrobial resistance (AMR) epidemic requires close observation of zoonotic antimicrobial resistance bacteria. The spread of these AMR infections to humans poses a concern to public health. Findings of the same kinds or clones in pig herds and humans working in these herds are among the several signs of LA-MRSA transmission from pigs to humans [3]. The frequency and length of farmers’ contact with cattle have been related to their carriage of LA-MRSA.

Humans were found to harbor livestock-associated MRSA strains in 17 of the 19 EU member states; including Holland 30.7%, 29.3% in Denmark and 9.7% in Spain which all had high LA-MRSA prevalence rates [4]. According to several research [5, 6], the majority of short-term farm visits only temporarily carry LA-MRSA, and only a small percentage (12%, 6%) remain positive after 24 h. Farmers’ families had a higher chance of having MRSA carriers in their households [7, 8]. Numerous animals, including pets and cattle that interact closely with people as well as wild animals, have been found to harbor *S. aureus* [9]. Methicillin-resistant *Staphylococcus aureus* (MRSA) associated with livestock (LA) is common. Methicillin-resistant *Staphylococcus aureus* (MRSA) associated with livestock is common in many animal species, with pigs being the most common host globally [10]. Simultaneously, there has been a rise in human cases, either with or without documented exposure to animals. This suggests that the production of livestock has a knock-on effect on the population, ultimately leading to illness in human [11].

According to Omoshaba et al. [12], animals have the ability to spread MRSA-resistant strains both horizontally and vertically, as well as through items meant for commercial processing and consumption. The number of countries reporting cases of MRSA pandemic strains is rising, and this indicates a serious risk to public health. To control cross-contamination and the rising trends in MRSA infections in humans and animals alike, an evaluation of the traits of MRSA from farm animals and humans who interact with them is still an essential step. Because of the MRSA’s intense circulation in the farming environment and society, the epidemiology is evolving dynamically [13].

In addition to spreading horizontally and vertically, animals can also spread MRSA-resistant strains through goods meant for commercial processing and consumption [12]. There is a serious risk to public health as more and more countries report experiencing outbreaks of MRSA pandemic strains. One of the most important steps in preventing cross-contamination and the rising trends in MRSA infections in humans and animals is to evaluate the traits of MRSA from farm animals and humans who come into contact with them. Due to extensive circulation in the farming community and community, the epidemiology of MRSA is dynamically changing [13]. Continual and sustained surveillance can provide information about emerging resistance or virulence. However, because a significant proportion of individuals remain asymptomatic, studies such as this is needed to evaluate the drug resistance pattern of these organisms.

## Methods

### Source of Samples

One hundred and twenty (180) Nasal and oral swab samples were obtained from goats, pigs and cattle. Also, swabs (nasal and palm) were collected from 48 different farm animals handlers across the different farms visited. The semi-intensive farming system was practiced at the farms. The farms practiced a secondary biosecurity system which was aimed at preventing pathogen spread within the farm. There were perimeter fences, closed passage way with clear warning notices for visitors, sterile showers and strict disinfection protocols. The study was conducted between September to November 2022 with reduced rainfall.

### Isolation of Organisms

Using the streak plate method, the samples were inoculated onto mannitol salt agar. The inoculated plates were incubated at 37°C for 24 h. After the period of incubation, bacterial colonies with golden yellow pigmentation on mannitol salt agar, characteristic of *S. aureus*, were subcultured onto newly made nutrient agar plates. The CLSI Guidelines (2024) were followed in the identification of the bacterial isolates as *S. aureus* based on their morphology, Gram staining, catalase characteristics, coagulase, and DNase tests.

### Antimicrobial Susceptibility Testing

The identified *S. aureus* isolates were tested for susceptibility to 10 different antimicrobial agents by the disc diffusion method on Mueller Hinton agar. Colonies from each isolate from an 18-hour old nutrient agar plate were introduced into sterile distilled water to create a bacterial suspension that met the 0.5 McFarland turbidity standard. A cotton swab was dipped into the bacterial inoculum and then pressed against the tube’s side to drain excess fluid. The same inoculum swab was then used to inoculate the whole surface of each Mueller Hinton agar plate, rotating the plate to guarantee confluent bacterial growth. The Mueller Hinton agar plates were pre-seeded with the isolates before the antibiotic discs were added.

After 24 h of incubation at 35^o^C, the plates were checked for zones of inhibition, measured using a ruler, and documented. After comparing the zones of inhibition produced by the antibiotics against the isolate with that of a reference guide provided by the [14], the isolates were classified as susceptible, resistant, or in intermediate status based on the zones of inhibition produced by the antibiotics against them.

### Detection of MRSA

Each isolate was suspended in sterile saline using the colonies from an overnight growth on nutrient agar plates. The turbidity of the solution was adjusted to 0.5 McFarland’s standard. A cotton swab was dipped into the bacterial mixture and then pressed against the tube’s side to drain excess fluid. The same inoculum swab was then used to inoculate the whole surface of each Mueller Hinton agar plate, rotating the plate to guarantee confluent bacterial growth. Cefoxitin disks were inserted into each Mueller-Hinton plate that had been inoculated. The plates were incubated at 37°C for 24 h. The zone surrounding the disc’s diameter was measured, and the findings were interpreted in accordance with CLSI guidelines. MRSA strains were identified among the isolates as those whose zone of inhibition was smaller than 22 mm. The strain of *Staphylococcus aureus* ATCC 25923 was employed as the control.

### Phenotypic Characterization of Biofilm Production in Congo Red Agar (CRA)

The isolates were cultivated on Congo red agar (CRA plates), which were made by combining 1 L of Brain Heart Infusion agar with 0.8 g of Congo red and 36 g of sucrose. After that, the plates were incubated at 37°C for 24 h. The ability of strains that produced slime (biofilm formers) to form rough black colonies served as a means of distinguishing them from strains of *S. aureus* that did not produce slime (red smooth colonies).

### Statistical Analysis

Prevalence rates of *S. aureus* strains from the different poultry animals were statistically analysed by T-test and results were considered significant at 95% confidence level.

## Results

**FIGURE 1:**
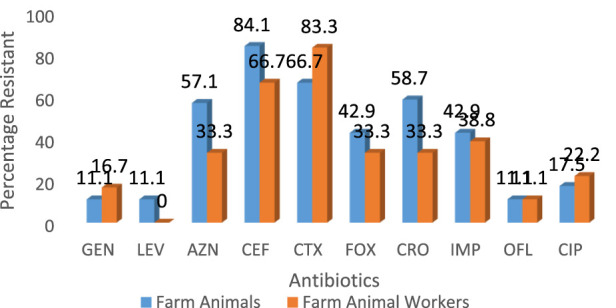
Comparison of the antibiotic resistance profile of *Staphylococcus aureus* isolates from livestock and breeders in Umuahia, Abia State Nigeria (Abia State, Nigeria, 2024).

A total of eighteen (37.5%) of *Staphylococcus aureus* were isolated from the different breeders whilst the breeding animals contributed a total of 63 isolates. The pigs had a larger proportion of the isolates amongst the animals (Table 1). Out of 60 cattle screened, 31.7% (19/60) harbored *S. aureus* in their nostrils. The same prevalence was noticed for goats. There was a statistically significant association (χ^2^ = 0.86; *p* = 0.83) between the prevalence of *S. aureus* and isolate source. In the same vein there was a significant association in the distribution of MRSA among breeders and farm animals (χ^2^ = 2.00; *p* = 0.37). Also, significant difference was observed in the biofilm Forming Potential of the *S. aureus* isolates from the poultry farms. Table 2 shows the distribution of *S. aureus* isolates according to their resistance to one, two or more antibiotics. Because *S. aureus* can spread to other people or emerge from colonized locations to cause infection, these levels of nasal carriage of the bacteria still pose a serious hazard to human health. The antibiotic susceptibility pattern of the isolates (Figure 1) showed that a greater percentage of the *S. aureus* isolates from the farm animal workers were susceptible to Levofloxacin, (83.3%), and Gentamicin (83.3%) followed closely by ciprofloxacin, (61.1%), ofloxacin, (77.8%). For the isolates obtained from the farm animals, imipenem, ofloxacin, levofloxacin, and gentamicin were the most potent antibiotics as the *Staphylococcus aureus* isolates showed 92.9%, 77.8%, 82.5% and 68.3% susceptibility respectively to the antibiotics. It can be noted from the Table that 28.0% of the pig isolates were resistant to at least one antibiotic. Seventeen out of the 81 isolates (20.9%) were resistant to one antibiotic, 31 out of the 81 isolates (38.2%) were resistant to two antibiotics while 48 out of the 81 isolates (59.3%) showed MDR. Twenty-six (41.3%) of the overall 63 *S. aureus* isolates obtained from the study animals were resistant to cefoxitin and considered to be methicillin resistant *S. aureus*. On the other hand, 6 (33.3%) out of the 18 *S. aureus* isolates from the breeders were Methicillin resistant (Table 3).

**TABLE 1:** Prevalence of *Staphylococcus aureus* isolated from breeders and farm animals in Umuahia, Abia State Nigeria (Abia State, Nigeria, 2024).

Isolate source	No of examined samples	Positive samples		χ-value	*p*-value
		No	%		
Pig	60	25	41.6	0.86	0.83
Goat	60	19	31.7		
Cattle	60	19	31.7		
Human	48	18	37.5		

**TABLE 2:** Distribution of the *Staphylococcus aureus* isolates according to their resistance to one, two or more antibiotics (Abia State, Nigeria, 2024).

Isolate source	No of isolates	Resistant to one antibiotic	Resistant to 2 antibiotics	MDR
Pig	25	7 (28.0)	11 (44.0)	18 (72.0)
Goat	19	3 (15.8)	5 (26.3)	9 (47.4)
Cattle	19	3 (15.8)	8 (42.1)	12 (63.1)
Human	18	4 (22.2)	7 (38.9)	8 (44.5)
Overall Resistance	81	17 (20.9)	31 (38.2)	48 (59.3)

**FIGURE 2:**
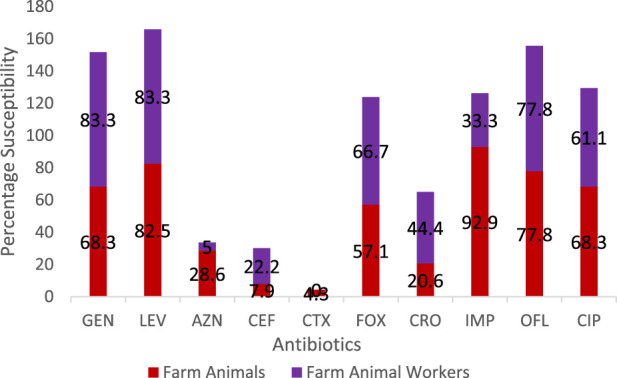
Comparison of the antibiotic susceptibility profile of *Staphylococcus aureus* isolates from livestock and breeders in Umuahia, Abia State Nigeria (Abia State, Nigeria, 2024).

**TABLE 3:** Distribution of methicillin-resistant Staphylococcus aureus among breeders and farm animals in Umuahia, Abia State Nigeria (Abia State, Nigeria, 2024).

Source of isolate	Total No of isolates	No (%) MRSA	No (%) MSSA	χ-value	*p*-value
Pig farmers	9	4 (44.4)	5 (55.6)		
Cattle farmers	6	2 (33.3)	4 (66.7)	2.00	0.37
Goat Farmers	3	0 (0.00)	3 (100)		
Total	**18**	**6 (33.3)**	**12 (66.7)**		
Goat	19	6 (31.5)	13 (68.4%)		
Cattle	19	8 (42.1)	11 (57.8)	1.20	0.54
Pig	25	12 (48.0)	13 (52.0)		
Total	**63**	**26 (41.3)**	**37 (58.7)**		

**TABLE 4:** Biofilm Forming Potential of the *Staphylococcus aureus* Isolates from Poultry farms in Umuahia Abia State, Nigeria (Abia State, Nigeria, 2024).

Source of isolate	Isolate status	Total No of positive of biofilm formation	No (%) negative to biofilm formation	χ-value	*p*-value
Goats	MRSA (n = 6)	4 (71.4)	2 (28.6)	1.30	0.25
	MSSA (n = 13)	5 (33.3)	8 (66.7)		
Cattle	MRSA (n = 8)	6 (75.0)	2 (25.0)	1.65	0.19
	MSSA (n = 11)	5 (45.4)	6 (54.5)		
Pigs	MRSA (n = 12)	7 (58.3)	5 (41.7)	0.98	0.32
	MSSA (n = 13)	5 (38.5)	8 (61.5)		
Overall Total	**63**	**32 (50.8)**	**31 (49.2)**		

MRSA, Methicillin Resistant *Staphylococcus aureus* * MSSA, Methicillin Susceptible *Staphylococcus aureus*.

## Discussion

As the global issue of antibiotic resistance worsens, the spread of MRSA from livestock farmers to their animals continues to be a serious public health concern. Genes that provide resistance to drugs can be significantly concentrated in livestock colonized by MRSA. One important epidemiological worry is the potential for these genes to be passed on.

The level of colonization by *S. aureus* was most prevalent among pig farm workers who had 56.2% colonization followed by cattle herders with 37.5% prevalence and least the goat farm workers with 18.7% prevalence. These prevalence levels have been reported across several related studies such as those by Khanna et al. [15], Effa et al. [16] and Locatelli et al. [17].

This study’s findings on the prevalence of *Staphylococcus aureus* colonization in goats (31.7%) are consistent with those of Pirzada et al. [18], who found that 38% of goats had sub-clinical mastitis. The current study findings are lower than those of Ali et al. [19] and Najeeb et al. [20], who reported prevalence of 47% and 53%, respectively. In addition, Raafat et al. [21] found that among goats living in freedom, the prevalence of *S. aureus* was lower (25.5%), and MRSA was significantly less common (1.38%) than it was in our study (31.5%). However, comparable results to this study’s have been found in other research that have examined the prevalence of MRSA in domestic goats [22, 23].

Chai et al. [24] and Murray et al. [25] have earlier identified similar antibiotic susceptibility characteristics with those of this study. It is noteworthy that there were differences in the isolates’ susceptibilities to gentamicin: the isolates from farm personnel were more susceptible (83.3%) than those from animals (68.3%). Nevertheless, others such as ofloxacin and levofloxacin had comparable susceptibility profiles. However, Azithromycin was more effective against the *S. aureus* isolated from the farm animals (28.6%) when compared to those from the workers (5.0%). Cefuroxime resistance was found to be higher in animal isolates (84.1%) compared to the farm personnel isolates (66.7%). Similarly, resistance to ciprofloxacin was 17.5% and 22.2%, respectively for the animals and workers. Equal level of resistance (11.1%) was observed for ofloxacin. The antibiogram pattern may indicate that the isolates could have a common ancestor. Thus, our work reports an increase in animal *S. aureus* isolate resistance to fluoroquinolones.

The isolates from the farm workers were observed to record higher resistance to cefotaxime, gentamicin and ciprofloxacin over their counterparts from the animals. Overall, there was increased resistance to ceftriaxone, cefotaxime and cefepime amongst all the isolates. The isolates’ resistance to ceftriaxone and cefotaxime is consistent with research conducted by Robert et al. [26]. LA-SA has been found in pigs [27, 28], poultry [29], sheep [30, 31], and goats [32], according to a number of investigations conducted worldwide. The results of this study are supported by [33] investigation, which likewise revealed increased resistance to cefepime at 94.7%. This could be because the MRSA strain contains intrinsically produced beta-lactamase.

Out of the 63 *S. aureus* isolates that were recovered from the research animals, 26 (41.3%) were found to be resistant to cefoxitin and were therefore considered to be methicillin-resistant *S. aureus*. However, Table 3 shows that 6 (33.3%) of the 18 *S. aureus* isolates from the livestock farmers were resistant to the antibiotic methicillin. Globally, *S. aureus* has become resistant to multiple drugs [34]. In comparison to the 59.3% seen in this study, Aydin et al. [35] revealed that 25.3% of *S. aureus* strains exhibited multidrug resistance. But Al-Ashmawy et al. [34] discovered a greater prevalence of multidrug resistance (MDR), with 84.1% of isolates showing high resistance levels, meaning they were resistant to three or more antibiotics.

Based on this investigation, it was found that 32 (or 50.8%) of the 63 *S. aureus* isolates that were isolated had the ability to produce biofilms. This trait might enable them to stick to various surfaces and avoid the effects of antibiotics. Two-thirds of illnesses are caused by bacteria found in biofilms, which exhibit a broad spectrum of antibiotic resistance. This observation aligns with the findings of Sharvari and Chitra [36] and Ramakrishna et al. [37], who reported higher resistance to routinely used antibiotics in staphylococci biofilm makers. Tuladhar [38] found higher values, with 78.4% of their isolates being strong biofilm makers. However, our results differed from other findings from Iran (93.1%) and Poland (99.2%) [39, 40].

### Conclusion

Antimicrobial resistance is a global problem of increasing proportions that we cannot afford to look away from. The 59.3% MDR observed in this study calls for swift actions in monitoring these organisms to contain further dissemination.

## References

[1] Anker J Koch A Ethelberg S Mølbak K Larsen J Jepsen MR. Distance to Pig Farms as Risk Factor for Community-Onset Livestock-Associated MRSA CC398 Infection in Persons Without Known Contact to Pig Farms–A Nationwide Study. Zoon Pub Health (2018) 65:352–60. 10.1111/zph.12441 PMC6115690 29314752

[2] Khairullah AR Sudjarwo SA Effendi MH Riwu KP Kurniawati DA Widodo A Pet Animals as Reservoirs for Spreading Methicillin-Resistant *S. aureus* to Human Health. J Adv Vet Anim Res (2023) 10:1–13. 10.5455/javar.2023.j641 37155545 PMC10122942

[3] Crombé F Argudín MA Vanderhaeghen W Hermans K Haesebrouck F Butaye P. Transmission Dynamics of Methicillin-Resistant *Staphylococcus aureus* in Pigs. Front Microbiol (2013) 4:57. 10.3389/fmicb.2013.00057 23518663 PMC3602589

[4] Kinross P Petersen A Skov R Van Hauwermeiren E Pantosti A Struelens MJ Livestock-Associated Methicillin-Resistant *S. aureus* (MRSA) Among Human MRSA Isolates. Eurosurveillance (2017) 22:16–00696. 10.2807/1560-7917.es.2017.22.44.16-00696 PMC5710135 29113628

[5] Frana TS Beahm AR Hanson BM Kinyon JM Layman LL Karriker LA Isolation and Characterization of Methicillin-Resistant *Staphylococcus aureus* from Pork Farms and Visiting Veterinary Students. PLoS One (2013) 8:e53738. 10.1371/journal.pone.0053738 23301102 PMC3536740

[6] Angen O Feld L Larsen J Rostgaard K Skov R Madsen AM Transmission of Methicillin- Resistant *Staphylococcus aureus* to Human Volunteers Visiting a Swine Farm. Appl Environ Microbiol (2017) 83:e01489-17–0. 10.1128/AEM.01489-17 28970219 PMC5691421

[7] Verkade E Bergh MK Benthem B Van-Cleef B van Rijen M Bosch T Transmission of Methicillin-Resistant *Staphylococcus aureus* CC398 From Livestock Veterinarians to Their Household Members. PLoS One (2014) 9:e100823. 10.1371/journal.pone.0100823 25062364 PMC4111304

[8] Liu W Liu Z Yao Z Fan Y Ye X Chen S. The Prevalence and Influencing Factors of Methicillin-Resistant *Staphylococcus aureus* Carriage in People in Contact with Livestock: A Systematic Review. Am J Infect Control (2015) 43:469–75. 10.1016/j.ajic.2014.12.009 25681305

[9] Monecke S Gavier-Widén D Hotzel H Peters M Guenther S Lazaris A Diversity of *Staphylococcus aureus* Isolates in European Wildlife. PLoS ONE (2016) 11:e0168433. 10.1371/journal.pone.0168433 27992523 PMC5161505

[10] Petinaki E Spiliopoulou I. Methicillin-Resistant *Staphylococcus aureus* Among Companion and Food-Chain Animals: Impact of Human Contacts. Clin Microbiol Infect (2012) 18:626–34. 10.1111/j.1469-0691.2012.03881.x 22550956

[11] Larsen J Petersen A Larsen AR Sieber RN Stegger M Koch A Emergence of Livestock-Associated Methicillin-Resistant *S. aureus* Bloodstream Infections in Denmark. Clin Infect Dis (2017) 00:1–5. 10.1093/cid/cix504 PMC5850567 28575216

[12] Omoshaba EO Ojo OE Oyekunle AM. Methicillin-Resistant *Staphylococcus aureus* Isolated From Raw Milk and Nasal Swabs of Small Ruminants in Abeokuta, Nigeria. Trop Anim Health Prod (2020) 52:2599–608. 10.1371/journal.pone.0053738 32451834

[13] Aires-de-Sousa M. Methicillin-Resistant *Staphylococcus aureus* Among Animals: Current Overview. Clin Microbiol Infect (2017) 23:373–80. 10.1016/j.cmi.2016.11.002 27851997

[14] CLSI. Clinical and Laboratory Standards Institute Performance Standards for Antimicrobial Susceptibility Testing (2024) 34th Edition.

[15] Khanna T Friendship R Dewey C Weese JS. Methicillin Resistant *S. aureus* Colonization in Pigs and Pig Farmers. Vet Microbiol (2008) 128(3-4):298–303. 10.1016/j.vetmic.2007.10.006 18023542

[16] Effa C Nicholas TKD Dayie S Akorli Y Anthony A. Epidemiology, Prevalence and Antibiotic Susceptibility Profiles of Methicillin-Resistant *Staphylococcus aureus* in Farm Animals and Farm Workers in the Central Region of Ghana. Afr J Microbiol (2018) 12(44):994–1003. 10.5897/ajmr2018.8993

[17] Locatelli C Cremonesi P Caprioli A Carfora V Lanzano A Barberio A Occurrence of Methicillin-Resistant *Staphylococcus aureus* in Dairy Cattle Herds, Related Swine Farms, and Humans in Contact With Herds. J Diary Sci (2017) 100(1):608–19. 10.3168/jds.2016-11797 27865508

[18] Pirzada M Malhi KK Kamboh AA Rind R Abro SH Lakho SA Prevalence of Subclinical Mastitis in Dairy Goats Caused by Bacterial Species. J Anim Health Prod (2016) 4(2):55–59.

[19] Ali Z Muhammad G Ahmad T Khan R Naz S Anwar H Prevalence of Caprine Sub-Clinical Mastitis, Its Etiological Agents and Their Sensitivity to Antibiotics in Indigenous Breeds of Kohat, Pakistan. Pak J Life Soc Sci (2010) 8(1):63–7.

[20] Najeeb MF Anjum AA Ahmad MU Khan HM Ali MA Sattar MM. Bacterial Etiology of Subclinical Mastitis in Dairy Goats and Multiple Drug Resistance of the Isolates. J Anim Plant Sci (2013) 23(6):1541–4.

[21] Raafat D Mrochen DM Al’Sholui F Heuser E Ryll R Pritchett-Corning KR Molecular Epidemiology of Methicillin-Susceptible and Methicillin-Resistant *Staphylococcus aureus* in Wild, Captive and Laboratory Rats: Effect of Habitat on the Nasal *S. aureus* Population. Toxins (2020) 12:80. 10.3390/toxins12020080 31991690 PMC7076793

[22] Kmet VC Uvalova A Stanko M. Small Mammals as Sentinels of Antimicrobial-Resistant Staphylococci. Folia Microbiol (Praha) (2018) 63:665–8. 10.1007/s12223-018-0594-3 29524153

[23] Gómez P González-Barrio D Benito D García JT Viñuela J Zarazaga M Detection of Methicillin Resistant *Staphylococcus aureus* (MRSA) Carrying the *mecC* Gene in Wild Small Mammals in Spain. J Antimicrob Chemother (2014) 69:2061–4. 10.1093/jac/dku100 24710026

[24] Chai M Muhammad ZS Amirah Huda KB Insyirah R Lennard ZL Pavitra M Methicillin-Resistant *Staphylococcus aureus* From Peninsular Malaysian Animal Handlers: Molecular Profile, Antimicrobial Resistance, Immune Evasion Cluster and Genotypic Categorization. Antibiotics (2022) 11:103–19. 10.3390/antibiotics11010103 35052980 PMC8773339

[25] Murray CJ Ikuta KS Sharara F Swetschinski L Aguilar GR Gray A Global Burden of Bacterial Antimicrobial Resistance in 2019: A Systematic Analysis. The Lancet (2022) 399(10325):629–55. 10.1016/s0140-6736(21)02724-0 PMC8841637 35065702

[26] Robert MR Allison DG Gilbert P. Resistance of Bacterial Biofilms to Antibiotics: A Growth-Rate Related Effect. J Antimicrob Chemother (2014) 22:777–80. 10.1371/journal.pone.0168433 3072331

[27] Pirolo M Gioffre A Visaggio D Gherardi M Pavia G Samele P Prevalence, Molecular Epidemiology, and Antimicrobial Resistance of Methicillin-Resistant *Staphylococcus aureus* From Swine in Southern Italy. BMC Microbiol (2019) 19:51. 10.1186/s12866-019-1422-x 30808302 PMC6390553

[28] Guo D Liu Y Han C Chen Z Ye X. Phenotypic and Molecular Characteristics of Methicillin-Resistant and Methicillin-Susceptible *Staphylococcus aureus* Isolated From Pigs: Implication for Livestock-Association Markers and Vaccine Strategies. Infect Drug Resist (2018) 11:1299–307. 10.2147/IDR.S173624 30197527 PMC6112776

[29] Mulders MN Haenen AP Geenen PL Vesseur PC Poldervaart ES Bosch T Prevalence of Livestock-Associated MRSA in Broiler Flocks and Risk Factors for Slaughterhouse Personnel in the Netherlands. Epidemiol Infect (2010) 138:743–55. 10.1017/S0950268810000075 20109255

[30] Giacinti G Carfora V Caprioli A Sagrafoli D Marri N Giangolini G Prevalence and Characterization of Methicillin-Resistant *Staphylococcus aureus* Carrying *mecA* or *mecC* and Methicillin-Susceptible *Staphylococcus aureus* in Dairy Sheep Farms in Central Italy. J Dairy Sci (2017) 100:7857–63. 10.3168/jds.2017-12940 28780098

[31] Carfora V Giacinti G Sagrafoli D Marri N Giangolini G Alba P Methicillin-Resistant and Methicillin-Susceptible *Staphylococcus aureus* in Dairy Sheep and In-Contact Humans: An Intra-farm Study. J Dairy Sci (2016) 99:4251–8. 10.3168/jds.2016-10912 27060817

[32] Chu C Yu CY Lee YH Su YC. Genetically Divergent Methicillin Resistant *Staphylococcus aureus* and Sec-Dependent Mastitis of Dairy Goats in Taiwan. BMC Vet Res (2012) 8:39. 10.1186/1746-6148-8-39 22455622 PMC3353860

[33] Shrestha B Pokhrel BM Mahopatra TM. Phenotypic Characterization of Nosocomial *S. aureus* Isolates in Special Reference to MRSA. J Infect Dev Ctries (2016) 3:554–60. 10.3855/jidc.474 19762974

[34] Al-Ashmawy MA Sallam KI Abd-Elghany SM Elhadidy M Tamura T. Prevalence, Molecular Characterization, and Antimicrobial Susceptibility of Methicillin-Resistant *Staphylococcus aureus* Isolated From Milk and Dairy Products. Foodborne Pathog Dis (2016) 13:156–62. 10.1089/fpd.2015.2038 26836943

[35] Aydin A Sudagidan M Muratoglu K. Prevalence of staphylococcal enterotoxins, toxin genes and genetic-relatedness of foodborne *Staphylococcus aureus* strains isolated in the Marmara Region of Turkey. Int J Food Microbiol (2011) 148 (2):99–106. 21652103 10.1016/j.ijfoodmicro.2011.05.007

[36] Sharvari S Chitra P. Evaluation of Different Detection Methods of Biofilm Formation in Clinical Isolates of Staphylococci. Int J Pharm Biol Sci (2012) 3(4):724–33.

[37] Ramakrishna P Syed A Ashthami V. Biofilm: Comparison Between the *Staphylococcus aureus* and Coagulase Negative *Staphylococcus* Species Isolated From a Rural Medical College Hospital in North Kerala, India. Int J Curr Microbiol Appl Sci (2014) 3(1):23–9.

[38] Tuladhar SH. Molecular Characterization of Regulatory Genes Associated With Biofilm Variation in a *Staphylococcus aureus* Strain. J Microbiol Biotechnol (2018) 18(1):28–34. 18239412

[39] Piechota MKB Frankowska-Maciejewska A Gruzewska A Wozniak-Kosek A. Biofilm Formation by Methicillin-Resistant and Methicillin-Sensitive *S. aureus* Strains From Hospitalized Patients in Poland. BioMedical Res Inst (2018) 465–7396. 10.1155/2018/4657396 PMC6327255 30687745

[40] Omidi MF Saffari M Sedaghat H Zibaei M Khaledi A (2020) Ability of Biofilm Production and Molecular Analysis of Spa and Ica Genes Among Clinical Isolates of Methicillin-Resistant *S. aureus. BMC Research Notes* 13, 1–9. 10.1186/s13104-020-4885-9 PMC6947956 31910883

